# Genotype Is Associated to the Degree of Virilization in Patients With Classic Congenital Adrenal Hyperplasia

**DOI:** 10.3389/fendo.2018.00733

**Published:** 2018-12-03

**Authors:** Vassos Neocleous, Pavlos Fanis, Leonidas A. Phylactou, Nicos Skordis

**Affiliations:** ^1^Department of Molecular Genetics, Function and Therapy, The Cyprus Institute of Neurology and Genetics, Nicosia, Cyprus; ^2^Cyprus School of Molecular Medicine, Nicosia, Cyprus; ^3^Division of Pediatric Endocrinology, Paedi Center for Specialized Pediatrics, Nicosia, Cyprus; ^4^St George's, University of London Medical School at the University of Nicosia, Nicosia, Cyprus

**Keywords:** Classic CAH, virilisation, CYP21A2, 21-hyrdroxylase deficiency, salt-wasting

## Abstract

**Background:** Molecular defects of *CYP21A2* consistently decrease 21-hydroxylase activity and result in a variable expression of disease severity in patients with congenital adrenal hyperplasia (CAH).

**Aim:** The genotype and biochemical findings were examined in an attempt to reveal any association to the degree of virilization in classic CAH patients.

**Methods:** The study included 18 CAH patients with complete characterization of *CYP21A2* mutations and were sorted based on the severity of the inherited mutations and the expected percentage of 21-hydroxylase enzyme activity.

**Results:** Eleven out of the 18 patients manifested the SW form with the remaining seven exhibiting the SV form. The most frequent genetic defect in the classic salt-wasting (SW) and simple virilising (SV) forms was the IVS2-13A/C>G (36.1%) mutation, followed by delEX1-3 (19.4%) and p.Ile172Asn (19.4%). Four patients, who shared a combination of two mutations belonging to the most severe type, manifested only the SW form. Four out of five patients who shared homozygosity in the IVS2-13A/C>G mutation, demonstrated the SW form and only one demonstrated the SV form. All four patients who shared the p.Ile172Asn mutation, either in the homozygous or compound heterozygous state, manifested the SV form. Interestingly, a female neonate with SW, bearing the IVS2-13A/C>G/Large del, exhibited complete male virilisation (Prader 5). The remaining four affected female new-borns also exhibited the SW form, with two of them virilised as Prader 3 and the other two as Prader 4. Virilisation with clitoromegaly was also observed in one female, who presented premature adrenarche and carried the least severe p.Pro30Leu mutation.

**Conclusion:** The frequency of the underlying mutations in our patients, with the classic form of CAH, varies but were quite similar to the ones reported in the Mediterranean region. Therefore, the identification of severe *CYP21A2* defects in Cypriot patients and their comparison with the incidence and severity in different populations, will create a valuable diagnostic tool for genetic counseling in the classic form of CAH.

## Introduction

Congenital adrenal Hyperplasia (CAH) is a group of autosomal recessive disorders caused by mutations in gene encoding enzymes, involved in cortisol biosynthesis and defective steroidogenesis (1). The most prevalent form of CAH is 21-hydroxylase (21-OH) (90–95% of cases) followed by the next most frequent type of 11β-hydroxylase (11β-OH) (~5% of cases) and other rarer types such as 17α-hydroxylase (17α-OH or 17,20-lyase), 3β-hydroxysteroid dehydrogenase type 2 (HSD3B2), steroidogenic acute regulatory protein (StAR), P450 cholesterol side-chain cleavage enzyme (SCC), P450 oxidoreductase (POR) and cytochrome b deficiency (CYB5A) (2, 3). 21-OH deficiency accounts for more than 95% of all CAH cases and is due to the molecular defects in the *CYP21A2* gene (4). The disorder has a broad spectrum of clinical phenotypes and severity depends on the patients' underlying *CYP21A2* genotype (5, 6). The deficiency is present in the course of fetal development and leads to varying degrees of prenatal virilisation of the external genitalia in affected girls. The clarification of the genetic background of CAH has been influential in the diagnosis and the classification of the disease (6, 7). Currently, the disorder is classified into the classic or non-classic (NC late onset) CAH form, respectively (8, 9). The classic form is further divided into the simple virilising (SV) form (~25% of individuals) and the salt-wasting (SW) form, in which aldosterone production is inadequate (≥75% of individuals). The patients are also divided into the SV and SW groups based on the presence of a milder allele. In the SV patients, excess androgen of the adrenals in the utero, result in genital virilization at birth in 46, XX females. In affected females, the excess androgens result in various degrees of enlargement of the clitoris, fusion of the labioscrotal folds, and formation of a urogenital sinus. Because the anti-müllerian hormone (AMH) is not secreted, the müllerian ducts develop normally into a uterus and fallopian tubes in affected females (10). Patients with the most severe SW classic form, are characterized by salt-wasting and the extremely low enzymatic activity of 21-OH. This leads to the deficiency of both aldosterone and cortisol usually accompanied by vomiting, dehydration, hypoglycaemia and hypotension as well as marked hyperkalaemia and hyponatraemia in the first weeks of after birth (11). Worldwide the estimated incidence of the classic form is 1:10,000 to 1:15,000, while the NC-CAH occurs in a frequency of 1:500 to 1:100 of live births and is estimated to be one of the most common autosomal recessive disorders (12–15).

Data from several new-born screenings and carrier analyses of the general population have estimated that the carrier incidence in the general population is 1:25–1:10 (16–18). Currently, more than 200 mutations in the *CYP21A2* gene, differing in prevalence and severity, have been reported and only 10 of them account for about 95% of the disease-causing alleles (6, 19).

Numerous studies have established a strong correlation between the genotype and the phenotype and over the last few decades mutation detection rates led to the identification of a large number of *CYP21A2* defects (20, 21). In this study, we present the molecular genetic features of the disease in patients with the classic form who are of Cypriot descent, over the last decade. Thus, the aim of this study is to describe a comprehensive *CYP21A2* mutation analysis in a cohort of classic CAH patients and to create a useful tool for clinicians and geneticists, necessary for the genetic diagnosis and management of not only Cypriot patients but also for international patients with 21-hydroxylase deficiency.

## Methods and results

### Patients and bioethics approval

From 2007 to 2018 18 patients of Greek Cypriot origin, with classic CAH, were phenotypically classified by one pediatric endocrinologist (N.S) based on clinical and hormonal criteria. Written and oral informed consent was obtained from the parents or guardians of the minors and all relatives screened for mutations in the *CYP21A2* gene. The project was approved by the Cyprus National Ethics Committee and all methods were performed in accordance with the relevant guidelines and regulations.

### Clinical, biochemical and genetic screening at diagnosis

All patients and their parents were genotyped and were categorized into the most severe SW form and the most severe SV form (Table 1). More specifically, patients with the SW form were initially allocated to this form based on clinical and biochemical findings of renal salt wasting (females with virilization at birth and males with vomiting, failure to thrive, hyponatremia, hyperkalemia, high plasma renin activity (PRA), and significantly high 17-OHP>75 nmol/L) in the first 2 weeks of life. The second group of patients categorized as having the SV form, also exhibited clinical symptoms of CAH without electrolyte imbalance (females with virilization at birth or later without any clinical evidence of salt loss at birth and males with clinical signs of sexual precocity with acceleration of growth and bone age, high 17-OHP > 75 nmol/L, and normal or slightly elevated PRA). The *CYP21A2* genes of the total number of patients who participated in the study were analyzed by Sanger DNA sequencing. The genetic investigation was done based on a cascade strategy as formerly described (18, 22). For the amplification of the 5′ untranslated region which is located in the first 167 nucleotides upstream of the ATG codon of the *CYP21A2* gene, the primers P1-P48 (23) were used to amplify a fragment of 370 bp. The 3' untranslated region that is 536 nucleotides downstream of the TGA stop codon of the *CYP21A2* gene was amplified using the primers: 5′AGATGCAGCCTTTCCAAGTG3′ and 5′AGCACAGTGGACCATCAGGT3′ (24). Multiplex ligation-dependent probe amplification (MLPA) technique (MRC Holland, Amsterdam, Netherlands) was used to detect any possible large gene deletions, duplications and large gene conversions in the *CYP21A2* gene of the patients under investigation, as previously described (22).

**Table 1:** The type of the molecular defects with clinical and biochemical data in the patients with Classic CAH.

	**Genotype**	**Form**	**Sex**	**Age of diagnosis**	**Clinical phenotype**	**17-OHP nmol/l basal**	**ACTH < 60 pg/ml**	Renin PRA^*^ ng/ml/hr(0.2–2.8)
1	IVS2-13A/C>G/IVS2-13A/C>G	SW	F	neonate	Ambiguous genitalia - Prader 3	>75.7	1450	10.3
2	IVS2-13A/C>G/IVS2-13A/C>G	SW	F	neonate	Ambiguous genitalia - Prader 3	>75.7	1355	9.4
3	IVS2-13A/C>G/Large del	SW	F	neonate	Ambiguous genitalia - Prader 5	>75.7	103	3.1
4	IVS2-13A/C>G/p.Gln318stop	SW	F	neonate	Ambiguous genitalia - Prader 4	>75.7	N/A	32.3
5	p.Phe306insT+p.Val281Leu/ p.Phe306insT+p.Val281Leu	SW	F	neonate	Ambiguous genitalia - Prader 4	>75.7	>2100	12
6	IVS2-13A/C>G/IVS2-13A/C>G	SW	M	neonate	Adrenal crisis	>75.7	>2100	11.4
7	IVS2-13A/C>G/IVS2-13A/C>G	SW	M	neonate	Adrenal crisis	>75.7	> 2100	10.7
8	IVS2-13A/C>G/del Exons 1_3	SW	M	neonate	Adrenal crisis	>75.7	2352	9.8
9	del Exons 1_3/del Exons 1_3	SW	M	neonate	Adrenal crisis	>75.7	>2100	8.5
10	del Exons 1_3/del Exons 1_3	SW	M	neonate	Adrenal crisis	>75.7	>2100	10.5
11	del Exons 1_3/p.Gln318stop	SW	M	neonate	Adrenal crisis	>75.7	1680	11.3
12	p.Pro30Leu/p.Pro30Leu	SV	F	6.5 years	Exaggerated premature clitoromegaly	>75.7	76.4	0.4
13	p.Ile172Asn/p.Ile172Asn	SV	F	neonate	Ambiguous genitalia at birth	>75.7	392	8.2
14	p.Ile172Asn/del of *CYP21A2*	SV	M	3 years	GnRH independent precocious puberty	>75.7	569	4.7
15	p.Ile172Asn/p.Ile172Asn	SV	M	5.0 years	GnRH independent precocious puberty	>75.7	38	4.7
16	p.Ile172Asn/p.Ile172Asn	SV	M	3.2 years	GnRH independent Precocious Puberty	>75.7	122	7.5
17	IVS2-13A/C>G/IVS2-13A/C>G	SV	M	5.5 years	GnRH independent precocious puberty	43.7	282	1.23
18	Partial conv with CYP21P:-4C>T, 92C>T, 118T>C, 138A>C/delEx 1_3	SV	M	6.5 years	GnRH independent precocious puberty	>75.7	N/A	N/A

PRA^*^, Plasma Renin Activity.

The type of molecular defects as well as the clinical and biochemical data of patients with classic CAH, are shown in Table 1. Eighteen patients with classic CAH were categorized in two groups (SW and SV) based on genotype/phenotype correlations (Table 1). More specifically, mutations allocated in the SW group resulted in no or minimal residual enzymatic activity (25–28). Mutations allocated to the SV group usually exhibited residual enzymatic activity of about 2% (29–31). The most severe form of CAH, the classic SW, was identified in 11 neonates (Table 1). Seven children with the SV form were also identified, at a median presentation age of 5 years (interquartile range (IQR) 3.2–6.5). The clinical presentation at diagnosis was considerably different between the SW and SV group (Table 1). All five females with SW CAH exhibited an expected electrolyte imbalance (hyponatremia, hypekalemia) and a variable degree of virilization of the external genitalia in accordance with the severity of mutations that they carried (Table 1).

All males with the SW CAH presented clinical signs of adrenal crisis, hyponatremia, hyperkalemia, dehydration, and hypovolemic shock. The children belonging to the SV group had no electrolyte imbalance. The girls with SV CAH presented ambiguous genitalia at birth and the boys manifested GnRH independent precocious puberty (Table 1).

The splice site mutation IVS2-13A/C>G in homozygosity was the most frequently detected genotype. Five out of 18 patients with the classic SW form of CAH were found in the homozygosity of the severe causing IVS2-13A/C>G splice mutant. The remaining 13 patients had a combination of compound heterozygote genotypes belonging to the most severe *null group* and the second most severe *group A* mutations as described in a previous study by our group (22). One patient affected with the SW form, was associated with the rare genotype p.Phe306insT+p.Val281Leu/p.Phe306insT+p.Val281Leu. The same genotype was detected both on the paternal and the maternal alleles.

Using the MLPA analysis, several deletions (DelEx1-3, del *CYP21A2*, Large del, 30 kb del) and a partial conversion (Partial conv with CYP21P:-4C>T, 92C>T, 118T>C, 138A>C) were identified. The DelEx1-3 was identified as the second most severe frequent defect and was detected in homozygosity or in the compound heterozygosity state in thirteen patients with various degrees of severity (Table 1).

**Figure 1:**
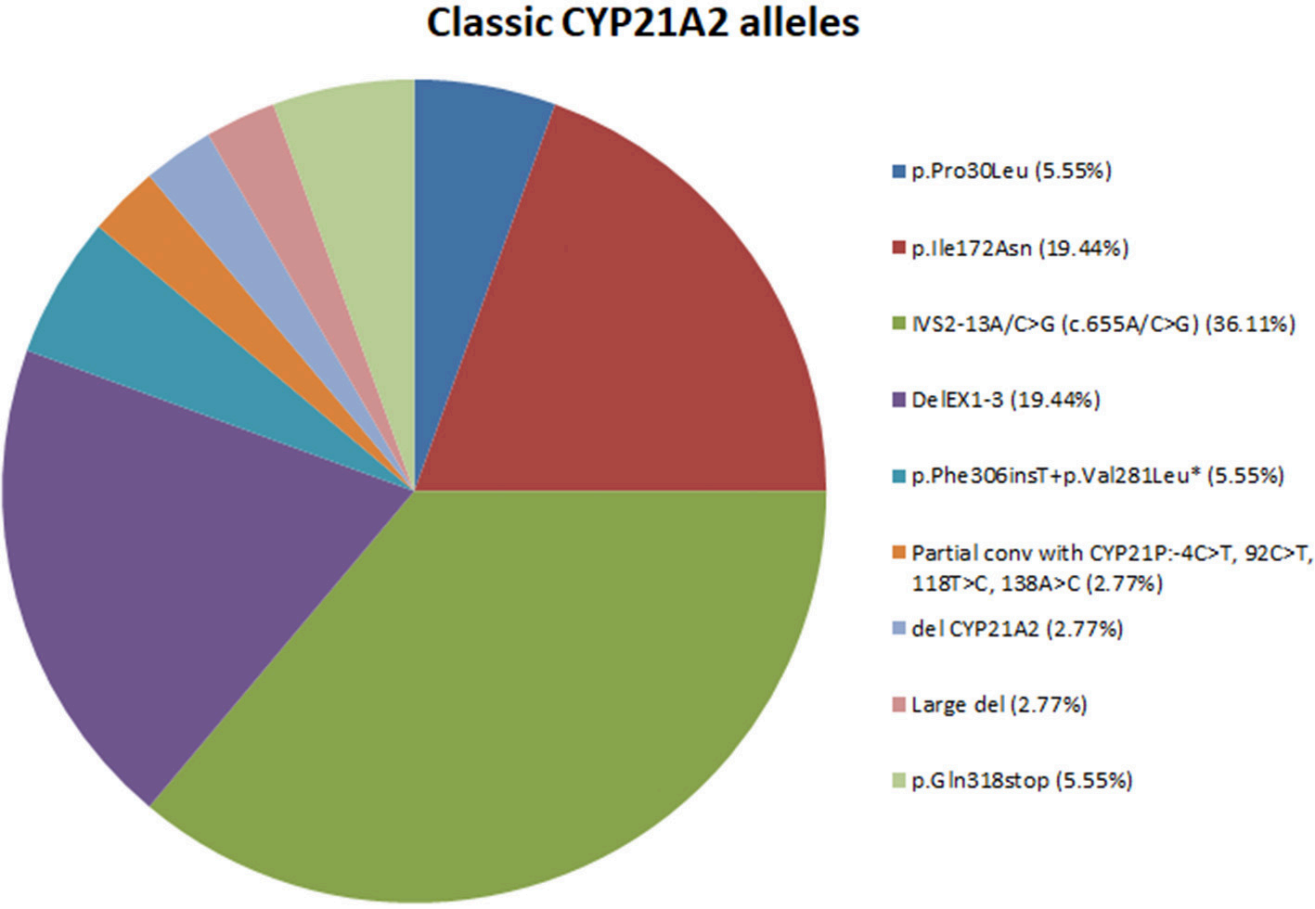
Pie-chart showing the percentage of mutations across the 18 classic CAH patients. *Mutation p.Phe306insT+p.Val281Leu fall under the category of multiple mutations because they are found *in cis* on the same allele.

In total, nine different variants were identified in the cohort of 18 patients with classic CAH and consisted of (a) two (22.22%) missense mutations, (b) one (11.11%) nonsense mutation, (c) one (11.11%) splicing mutation, (d) one (11.11%) frameshift mutation, (e) one (11.11%) partial conversion, and finally (f) three (33.33%) large deletions. The overall frequency of the identified molecular defects detected in our patients is also depicted in Figure 1. In the 36 non-related alleles, the most frequent mutation was IVS2-13A/C>G (36.11%) followed by DelEx1-3 (19.44%). A series of seven other less frequent and mostly severe mutations were identified and are also depicted in Figure 1.

## Discussion

Our data represent a comprehensive portrayal of the classic clinical forms of CAH over a period of time in Cyprus. From 2007 to 2017, 18 patients with either the SW or SV form of CAH were categorized and genotyped at the Molecular Genetics, Function and Therapy (MGFT) department of the Cyprus Institute of Neurology and Genetics. With an estimated population of 701,000 Greek Cypriots (Cyprus statistical service 2016_ http://www.mof.gov.cy/mof/cystat/statistics.nsf/populationcondition_21main_puparchive_en/populationcondition_21main_puparchive_en?OpenForm&yr=2016) and the recent report by our group that the true carrier frequency of *CYP21A2* in Greek-Cypriots is 1:10 (18), the CAH prevalence is therefore predicted to be around ~1750 (701,000 × ½ × ½ × ^1^/10 × ^1^/10 = 1752.5). Subsequently, the current 18 classic CAH patients identified by our group make up only a 1.03% of the total of CAH cases projected (~1,750) to exist in the Greek Cypriot population. Prompt screening in combination with *CYP21A2* genetic analyses, enables clinicians to manage severe cases in the neonatal period promptly, even before the appearance of any electrolyte imbalance and/or urgent adrenal crisis.

As expected, the clinical presentation of our classic CAH patients showed a spectrum of phenotypes and as demonstrated from the current and previous studies, the clinical presentation was substantially different between the SW and SV groups (22, 32). The female neonates with SW presented a variable degree of virilization in accordance with the severity of the genetic defect accompanied by hyponatremia and hyperkalemia, whereas all males exhibited signs of adrenal crisis (electrolyte imbalance and hypovolemic shock). Interestingly, one of the five female neonates with SW had an external genital appearance of Prader 5, with the remaining four classified as Prader 3 or 4. The neonate of our study with SW and the external genital appearance of Prader 5, were carried in the compound heterozygote state, the IVS2-13A/C>G and a large deletion. Several recent and older reports have shown that complete deletion of *CYP21A2* in Caucasians changes the genomic organization in the RCCX module to the status of C4A-CYP21A1P-TNXA/TNXB (21, 33). To date, at least nine kinds of chimeric TNXA/TNXB genes have been identified and associated with Ehlers-Danlos syndrome as well as with CAH (33). This combination of the IVS2-13A/C>G with a large deletion has been associated with the most severe SW phenotype (33–36). This phenotype is part of a group of chimeras and is common among CAH patients of Caucasian origin and has been referred to as a classic or common type of chimera (37). None of the children in the SV group had any electrolyte imbalance as expected. All males belonging to the SV group exhibited GnRH independent precocious puberty (pubic hair, penile increase, pre pubertal testes) at different ages.

Currently, more than 200 mutations in the *CYP21A2* gene have been described in several studies and there is a good correlation between the clinical phenotype and the patient genotypic findings (1, 21, 38–43). In general, our genotype-phenotype correlation was in accordance with previous studies and showed a positive predictive value for patients carrying mutations belonging to the null group (44–46). Patients carrying the supposedly milder mutation p.Pro30Leu, have previously been reported to demonstrate poor genotype-phenotype correlation and showed a divergence between the observed and predicted phenotype (6, 46). In a similar fashion a female patient from our cohort, homozygous for p.Pro30Leu, was clinically and biochemically identified with the SV form, with a premature pubarche clitoromegaly at 6.5 yrs. It is possible that other genetic variation(s) might also exist in other genes known to be implicated in the salt balance of CAH, for this specific female, that carried a homozygosity p.Pro30Leu. Such candidate genes where variations have been reported to exist are the *CYP2C19* and *CYP34A3* (47). Therefore, the infrequent phenomenon of digenic inheritance (DI), where the patients co-inherit biallelic or even triallelic mutations in two distinct genes (48, 49), *in cis* or *in trans*, and are sufficient to cause pathology with a usually defined and severe diagnosis, could also be the case with the female patient of our cohort carrying the homozygosity p.Pro30Leu.

According to genetic findings from previous studies as well as our present study, 17 different variants have been identified in the Greek-Cypriot population and are scattered throughout the entire coding sequence of the *CYP21A2* gene (22, 32, 40, 42, 43). In the present study we identified nine different variants and the most frequent defect among the 36 tested alleles was the IVS2-13A/C>G (36.11%) followed by DelEx1-3 (19.44%) and a series of seven other less frequent mutations.

In conclusion, the pathogenesis and the clinical presentation of the classic CAH depend on the severity of the underlying *CYP21A2* gene defects. Our study describes the complexities encountered in patients with classic CAH. The genotypic analysis of our patients with classic CAH confirmed their diagnosis in one of the two main forms of the disease, with an exceptional genotype-phenotype correlation. Knowing about the genetic defects will be valuable in the antenatal diagnosis, management and genetic counseling of existing and future families affected by these gene defects.

## Author contributions

All authors listed have made a substantial, direct and intellectual contribution to the work, and approved it for publication.

### Conflict of interest statement

The authors declare that the research was conducted in the absence of any commercial or financial relationships that could be construed as a potential conflict of interest.
